# Macular perfusion changes in people administered two types of COVID-19 vaccines: optical coherence tomography angiography study

**DOI:** 10.5935/0004-2749.2023-0051

**Published:** 2024-03-05

**Authors:** Zeynep Serikoglu Akbas, Tuna Ozan, Ceyhun Arici

**Affiliations:** 1 Department of Ophthalmology, Cerrahpasa Medical Faculty, Istanbul University- Cerrahpasa, Istanbul, Turkey; 2 Department of Ophthalmology, Beyoglu Eye Training and Research Hospital, University of Health Sciences Turkey, Istanbul, Turkey

**Keywords:** Tomography, optical coherence, Angiography, COVID-19 vaccines, COVID-19, Coronavirus infections, SARS-CoV-2, mRNA vaccines, CoronaVac, Incidence

## Abstract

**Purpose:**

To evaluate macular chorioretinal flow changes on optical coherence tomography angiography, in participants who received inactivated and messenger RNA (mRNA) vaccines to prevent Coronavirus disease 2019 (COVID-19).

**Methods:**

In this prospective cohort study, healthy participants who received two doses of an inactivated COVID-19 vaccine (CoronaVac) and then one dose of an mRNA vaccine (BNT162b2) were examined before and after each vaccination. Ophthalmologic examination and imaging with optical coherence tomography angiography were performed during each visit. We evaluated vascular densities in the superficial and deep capillary plexuses in foveal, parafoveal, and perifoveal areas; the foveal avascular zone; and choriocapillaris flows (in 1- and 6-mm-diameter areas).

**Results:**

One eye in each of the 24 participants was assessed. Superficial capillary plexus vascular densities in the parafoveal area were significantly lower after the second dose of the CoronaVac vaccine than after the first dose. In the deep capillary plexus, vascular attenuation was observed only in the parafoveal region after the first CoronaVac dose. However, in all regions, the deep capillary plexus vascular densities and subfoveal choriocapillaris flow were significantly decreased after the second CoronaVac dose. After the BNT162b2 dose, the superficial capillary plexus vascular densities, the deep capillary plexus vascular densities, and subfoveal choriocapillaris flow of most regions were significantly lower than those before vaccinations.

**Conclusion:**

Vascular attenuation, observed particularly after the second dose of the CoronaVac vaccine, may explain the pathogenesis of postvaccine ocular ischemic disorders reported in the literature. However, these disorders are extremely rare, and the incidence of thrombotic events caused by COVID-19 itself is higher.

## INTRODUCTION

The outbreak of Coronavirus disease 2019 (COVID-19), caused by the severe acute respiratory syndrome Coronavirus 2 (SARS-CoV-2), has prompted global intensive research to combat the disease through vaccine development. Many effective vaccines have been developed during the emergency period, including messenger RNA (mRNA) vaccines (e.g., BNT162b2 [BioNTech, Pfizer, New York City, NY, USA)] and mRNA-1273 [Moderna, Cambridge, MA, USA]); protein subunit vaccines (e.g., NVX-CoV2373; Novavax, Gaithersburg, MD, USA); vector vaccines (e.g., Ad26.COV2 [Janssen Pharmaceutical Companies, Beerse, Belgium] and ChAdOx1 nCoV-19 [AZD1222; Oxford University, Oxford, UK, and AstraZeneca, Cambridge, UK]), and entire virus vaccines (e.g., PiCoVacc [CoronaVac 19; Sinovac Biotech Ltd., Beijing, China] and BBIBP-CorV, [Sinopharm, Beijing, China])^(1)^. However, the efficacy and safety of these vaccines have been debated intensively. Immune-mediated adverse events and vaccine-induced thrombotic events have been reported. However, adverse ocular effects of COVID-19 vaccines have rarely been reported^(2,3)^.

In this prospective cohort study, we used optical coherence tomography angiography (OCTA) to examine the effects of two doses of an inactivated virus vaccine (CoronaVac), followed by one dose of an mRNA vaccine (BNT162b2), on macular vascular parameters in healthy individuals.

## METHODS

Only high-quality images, with an image quality index of >0.6, were included. Of 27 potential participants, three were excluded because of poor OCTA image quality. We also excluded individuals with systemic diseases, such as diabetes mellitus, hypertension, and vascular diseases, as well as those who had undergone previous ocular surgery and who had media opacities, ocular conditions such as uveitis, glaucoma, and refractive error (in spherical equivalents) greater than ±3.00 diopters.

The 24 participants received two doses of the CoronaVac vaccine 4 weeks apart, on average, and one dose of the BNT162b2 vaccine 18 weeks later, on average. Before vaccination and within 2-4 weeks after each dose of vaccine, each participant underwent a complete ophthalmological examination, in which the best- corrected visual acuity and intraocular pressure were measured and biomicroscopic and fundoscopic assessments were conducted. OCTA was performed in one eye of each participant with the Angiovue system (RTVue XR Avanti, Software Version 2016.2.0 with DualTrac; Optovue, Inc.). Images with poor signal quality (signal strength of ≤6/10) were not used.

OCTA images of one eye of each of 16 unvaccinated healthy participants were included as a control group to test the reproducibility of the measurements taken by the Angiovue system (Table 1). The OCTA imaging was performed twice within a 3-week interval. This interval was used because antibody formation peaked in the third week. The same macular vascular parameters on the two images were compared.

**Table 1:** Mean age and sex distribution for study and control groups

Characteristic	Study group	Control group	p-value
Age (years)	34.7 ± 7.8	41.3 ± 9.91	0.084
Gender (male/female)	7/17	5/11	

In addition, 6 6 mm retinal angiographic images were obtained with the AngioVue system. The OCTA scan covered the areas within three concentric circles with 1-, 3-, and 6-mm diameters, which represented the foveal, parafoveal, and perifoveal regions, respectively. The software automatically estimated the percentage of the area occupied by vasculature (vascular density) that was within the central 1-mm foveal center and the 1- to 3-mm rim of the parafoveal area. These calculations included the vascular densities of the superior and inferior hemifield parafoveal zones and the temporal, superior, nasal, and inferior zones. We also measured vascular densities in the outer 3- to 6-mm rim (the perifoveal zone).

Superficial and deep capillary plexus (SCP and DCP) vascular densities were calculated automatically by the Angiovue system, and the results were expressed in percentages. Choriocapillaris flows were calculated manually, whereby circular areas 1 mm in diameter represented the foveal area, and circular areas 6 mm in diameter represented the macular area (to correspond to the circles in retinal imaging). The circular areas were drawn manually. We calculated the percentage of the flow within a selected area, which was evaluated as the percentage of choriocapillaris flow.

We used the OCTA scans to evaluate the foveal avascular zone area and the SCP and DCP vascular densities in the foveal, parafoveal, and perifoveal zones; areas 1 and 6 mm in diameter were used to calculate the flow distribution in the choriocapillaris for each patient at each visit. A split-spectrum amplitude-decorrelation angiographic software algorithm was used to construct a flow map of each scan. The motion correction technology incorporated in the Optovue software was used to compensate for motion artifacts.

### Statistical analysis

To perform the data analysis, we used IBM SPSS for Windows, version 22 (IBM Corporation, Armonk, NY, USA). Descriptive statistics were calculated as means and standard deviations, minimums, and maximums for continuous data, and as frequencies and proportions for categorical data. We used the Shapiro-Wilk test to evaluate the normality of the data and the conformity of the numerical variables to the normal distribution. Values of variables were calculated as means and standard deviations. We used the Wilcoxon test to compare older and newer measurements. The results were evaluated within a 95% confidence interval, and p-values of <0.05 were considered statistically significant.

The four measurement values obtained for each subject (baseline, after the first and second CoronaVac doses, and after the BNT162b2 dose) were compared in pairs.

## RESULTS

The study group included 7 men and 17 women, and the control group included 5 men and 11 women. The mean ages of the two groups were 34.7 and 41.3, respectively (Table 1).

The SCP vascular densities in the parafoveal region after the second dose were decreased significantly in comparison with after first dose measurements (p=0.034; Table 2). No significant changes were detected in the foveal avascular zone.

**Table 2:** Pairwise comparison of measurements of superficial capillary plexus vascular densities and foveal avascular zones

	Superficial capillary plexus vascular density (%)		
Comparison	Measurement (1): mean (SD)	Measurement (2): mean (SD)	p-value
**Fovea**			
(1) Baseline and (2) after first CoronaVac dose	22.31 (6.570)	22.01 (6.308)	0.561
(1) Baseline and (2) after second CoronaVac dose	20.94 (7.304)	20.07 (6.941)	0.255
(1) Baseline and (2) after BNT162b2 dose	21.89 (8.225)	26.80 (31.410)	0.410
(1) After first CoronaVac dose and (2) after second CoronaVac dose	22.26 (5.885)	21.43 (5.564)	0.251
(1) After first CoronaVac dose and (2) after BNT162b2 dose	23.36 (6.411)	28.00 (27.919)	0.347
(1) After second CoronaVac dose and (2) after BNT162b2 dose	20.17 (7.923)	29.21 (35.048)	0.235
**Parafovea**			
(1) Baseline and (2) after first CoronaVac dose	53.77.(3.862)	54.01 (3.481)	0.747
(1) Baseline and (2) after second CoronaVac dose	53.80 (3.904)	52.27 (5.199)	0.168
(1) Baseline and (2) after BNT162b2 dose	54.76 (3.330)	52.69 (4.142)	**0.004**
(1) After first CoronaVac dose and (2) after second CoronaVac dose	54.21 (2.793)	52.11 (5.315)	**0.034**
(1) After first CoronaVac dose and (2) after BNT162b2 dose	53.34 (3.387)	51.90 (4.082)	**0.047**
(1) After second CoronaVac dose and (2) after BNT162b2 dose	52.38 (5.304)	53.43 (3.911)	0.414
**Perifovea**			
(1) Baseline and (2) after first CoronaVac dose	52.03 (3.295)	51.70 (3.058)	0.588
(1) Baseline and (2) after second CoronaVac dose	51.96 (3.267)	50.80 (2.882)	0.101
(1) Baseline and (2) after BNT162b2 dose	52.48 (3.109)	50.80 (3.407)	**0.007**
(1) After first CoronaVac dose and (2) after second CoronaVac dose	51.82 (2.460)	50.83 (2.939)	0.078
(1) After first CoronaVac dose and (2) after BNT162b2 dose	51.04 (3.220)	50.40 (3.308)	0.212
(1) After second CoronaVac dose and (2) after BNT162b2 dose	50.80 (3.060)	51.43 (3.224)	0.407
**Foveal avascular zone (mm^2^)**			
(1) Baseline and (2) after first CoronaVac dose	0.2618 (0.08010)	0.2632 (0.08045)	0.647
(1) Baseline and (2) after second CoronaVac dose	0.2763 (0.09604)	0.2765 (0.09089)	0.970
(1) Baseline and (2) after BNT162b2 dose	0.2683 (0.09421)	0.2704 (0.09460)	0.592
(1) After first CoronaVac dose and (2) after second CoronaVac dose	0.2694 (0.08504)	0.2698 (0.08533)	0.952
(1) After first CoronaVac dose and (2) after BNT162b2 dose	0.2350 (0.08612)	0.2359 (0.08156)	0.700
(1) After second CoronaVac dose and (2) after BNT162b2 dose	0.2724 (0.09870)	0.2757 (0.10030)	0.418

The first and second measurement columns in the table list the two values corresponding to the two times listed in the left column. For example, under “Fovea,” the first measurement value for “(1) Baseline and (2) after BNT162b2 dose” corresponds to the mean value of the baseline measurements (21.89), and the second measurement value corresponds to the mean value of the post-BNT162b2 measurements (26.80).

SD = standard deviation.

* Boldface values are significant.

**Figure 1:**
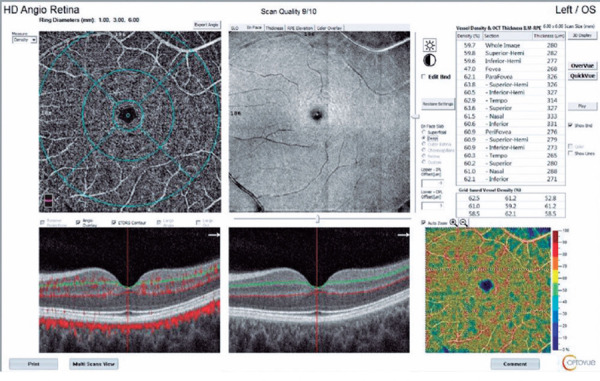
Optical coherence tomography angiography (OCTA) showing a participant’s deep capillary plexus before vaccinations. The enface images at the top, and cross-sectional retinal images are seen at the bottom. In the upper right table, numerical vascular density percentages are given according to the regions of the macula.

The DCP vascular densities decreased in all regions after the first dose. This decrease was statistically significant only in the parafovea (p=0.041; Table 3). We observed general vascular attenuation after the second dose, in comparison with the first dose, and this change was significant in the foveal region (p=0.04). A cumulative significant vascular reduction from baseline was observed after the second dose in the parafoveal (p=0.033) and perifoveal areas (p=0.049; Figures 1 2).

**Table 3:** Pairwise comparison of measurements of deep capillary plexus vascular densities

	Deep capillary plexus vascular density (%)		
Comparison	Measurement (1): mean (SD)	Measurement (2): mean (SD)	p-value*
**Fovea**			
(1) Baseline and (2) after first CoronaVac dose	39.99 (7.046)	39.83 (6.402)	0.769
(1) Baseline and (2) after second CoronaVac dose	38.76 (7.703)	37.47 (6.767)	0.116
(1) Baseline and (2) after BNT162b2 dose	39.93 (7.779)	38.64 (7.440)	**0.021**
(1) After first CoronaVac dose and (2) after second CoronaVac dose	40.14 (6.182)	38.70 (5.791)	**0.040**
(1) After first CoronaVac dose and (2) after BNT162b2 dose	41.56 (6.130)	40.83 (5.866)	0.152
(1) After second CoronaVac dose and (2) after BNT162b2 dose	38.10 (7.368)	39.10 (7.720)	0.218
**Parafovea**			
(1) Baseline and (2) after first CoronaVac dose	58.89 (4.162)	57.06 **(**5.094)	**0.041**
(1) Baseline and (2) after second CoronaVac dose	58.72 (4.100)	56.41 (3.498)	**0.033**
(1) Baseline and (2) after BNT162b2 dose	59.57 (3.136)	56.05 (3.567)	**0.000**
(1) After first CoronaVac dose and (2) after second CoronaVac dose	56.48 (5.069)	56.02 (3.861)	0.614
(1) After first CoronaVac dose and (2) after BNT162b2 dose	56.03 (5.118)	54.95 (3.873)	0.256
(1) After second CoronaVac dose and (2) after BNT162b2 dose	56.15 (3.921)	56.36 (3.264)	0.818
**Perifovea**			
(1) Baseline and (2) after first CoronaVac dose	57.87 (6.998)	55.35 (7.297)	0.057
(1) Baseline and (2) after second CoronaVac dose	57.54 (6.923)	54.16 (5.570)	**0.049**
(1) Baseline and (2) after BNT162b2 dose	58.72 (5.552)	53.34 (4.520)	**0.000**
(1) After first CoronaVac dose and (2) after second CoronaVac dose	54.59 (7.154)	53.67 (5.947)	0.468
(1) After first CoronaVac dose and (2) after BNT162b2 dose	53.67 (7.312)	51.95 (4.928)	0.180
(1) After second CoronaVac dose and (2) after BNT162b2 dose	53.50 (6.120)	53.94 (4.121)	0.756

The first and second measurement columns in the table list the two values corresponding to the two times listed in the left column. For example, under “Fovea,” the first measurement value for “(1) Baseline and (2) after BNT162b2 dose” corresponds to the mean value of the baseline measurements (39.93), and the second measurement value corresponds to the mean value of the post-BNT162b2 measurements (38.64).

SD = standard deviation.

* Boldface values are significant.

**Figure 2:**
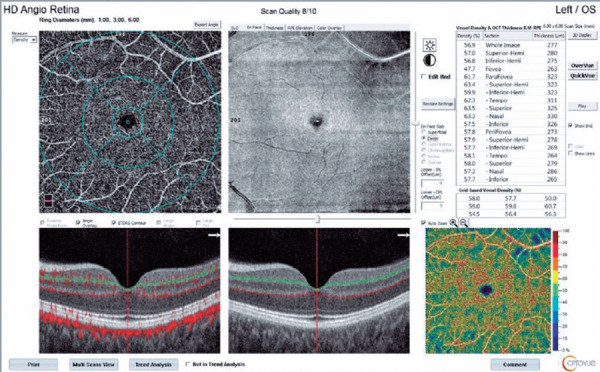
Optical coherence tomography angiography (OCTA) showing a participant’s deep capillary plexus after vaccinations.

In the 1-mm-diameter subfoveal area, choriocapillaris flow decreased significantly between baseline and the second dose (p=0.000; Table 4) and between the first and second doses (p=0.036).

**Table 4:** Pairwise comparison of measurements of choriocapillaris flow

Comparison	Measurement (1): mean (SD)	Measurement (2): mean (SD)	p-value*
**1-mm-Diameter area**			
(1) Baseline and (2) after first CoronaVac dose	71.88(3.622)	70.71 (4.250)	0.084
(1) Baseline and (2) after second CoronaVac dose	72.36 (3.296)	69.45 (3.507)	**0.000**
(1) Baseline and (2) after BNT162b2 dose	71.19(3.369)	68.18 (4.698)	**0.000**
(1) After first CoronaVac dose and (2) after second CoronaVac dose	70.81 (4.314)	69.41 (3.612)	**0.036**
(1) After first CoronaVac dose and (2) after BNT162b2 dose	70.35 (4.274)	69.34.(4.767)	0.182
(1) After second CoronaVac dose and (2) after BNT162b2 dose	68.49 (3.585)	68.04 (4.884)	0.628
**6-mm-Diameter area**			
**(1)** Baseline and (2) after first CoronaVac dose	71.21 (2.885)	70.71 (3.080)	0.253
(1) Baseline and (2) after second CoronaVac dose	71.45(2.459)	71.40(2.984)	0.899
(1) Baseline and (2) after BNT162b2 dose	70.89 (2.793)	70.57(3.118)	0.618
(1) After first CoronaVac dose and (2) after second CoronaVac dose	70.84(3.150)	71.40(2.870)	0.088
(1) After first CoronaVac dose and (2) after BNT162b2 dose	71.39(3.186)	71.64(2.940)	0.573
(1) After second CoronaVac dose and (2) after BNT162b2 dose	71.06(3.091)	70.90 (3.038)	0.808

The first and second measurement columns in the table list the two values corresponding to the two times listed in the left column. For example, under “1-mm-Diameter area,” the first measurement value for “(1) Baseline and (2) after BNT162b2 dose” corresponds to the mean value of the baseline measurements (71.19), and the second measurement value corresponds to the mean value of the post-BNT162b2 measurements (68.18).

SD = standard deviation.

* Boldface values are significant.

We observed no significant difference between the measurements after the second CoronaVac dose and after the BNT162b2 dose. We did observe significant decreases in SCP vascular densities in the parafoveal and perifoveal regions, in the DCP vascular densities in all macular regions, and in subfoveal choriocapillaris flow between measurements at baseline and after the BNT162b2 dose. In the control group, we found no significant differences in any parameter in the repeated measurements (Table 5).

**Table 5:** Comparison of the measurements of the unvaccinated healthy control group

Location	First measurement: mean (SD)	Second measurement: mean (SD)	p-value
**SCP VD (%)**			
Fovea	21.03 (8.97)	21.01 (8.88)	0.914
Parafovea	54.47 (2.84)	54.60 (2.70)	0.701
Perifovea	52.46 (3.4)	52.49 (3.37)	0.796
**DCP VD (%)**			
Fovea	39.57 (8.47)	39.73 (8.49)	0.860
Parafovea	59.33 (3.48)	59.31 (3.37)	0.800
Perifovea	58.61 (5.50)	58.65 (5.45)	0.651
**Foveal avascular zone (mm^2^)**	0.273 (0.11)	0.272 (0.07)	0.789
**Choriocapillaris flow (%)**			
1-mm-Diameter area	67.32 (3.65)	67.32 (3.63)	0.701
6-mm-Diameter area	70.25 (2.70)	70.25 (2.73)	0.773

DC= deep capillary plexus; SCP= superficial capillary plexus; S= standard deviation; VD= vascular density.

## DISCUSSION

Vaccines administered to large populations to prevent COVID-19 may have adverse effects^(4)^. Of the systemic or ophthalmological adverse effects of the vaccines reported in the literature, many are allergic, immune-mediated, ischemic, or thrombotic^(5,6)^.

Ocular findings are observed both after COVID-19 and after administration of mRNA or inactivated COVID-19 vaccines^(3)^. These findings include anterior segment symptoms and disorders, such as conjunctivitis, Chemosis, discharge, and epiphora, and posterior segment involvement, as in retinal hemorrhage, cotton wool spots, hyperreflective lesions as noted on optical coherence tomography, central retinal artery/vein occlusion, paracentral acute middle maculopathy, acute macular neuroretinopathy (AMN), and acute ischemic optic neuropathy during and after COVID-19^(7,8)^.

Turker et al. demonstrated a significant vascular reduction in two quadrants of the parafoveal SCP and all quadrants of the DCP on OCTA images of patients who recovered from COVID-19, in comparison with healthy controls^(9)^. Cennamo et al. examined OCTA images of 40 patients 6 months after recovery from COVID-19 and reported significant vascular attenuation in the entire SCP, entire DCP, foveal regions, and parafoveal regions in comparison with the healthy controls^(10)^. These two studies provided valuable OCTA evidence that COVID-19 has effects on retinal vascularization. It is possible that the infection itself and the vaccines trigger similar immunological or ischemic events through mechanisms such as molecular mimicry.

Although the causality has not been established, some adverse ocular effects have been reported after vaccination against COVID-19. Ng et al. documented adverse ophthalmological effects from various COVID-19 vaccines. In their review, most of the ocular disorders reported were immune-mediated, vascular, or ischemic in nature. The number of possible ophthalmological cases reported after mRNA and vector vaccines have increased since the vaccines first became available^(3)^. This increase may have occurred because such vaccines are being administered to large populations worldwide.

Although thrombotic and ischemic adverse effects have been reported after receipt of COVID-19 vaccines, these are extremely rare. They may have resulted from molecular mimicry of the vaccine or production of antigen-specific cells, and they may be antibody-mediated hypersensitivity reactions^(11)^. One study showed that the incidence of portal and cerebral vein thrombosis is higher among patients with COVID-19 than in people who have received COVID-19 vaccines^(12)^.

To elucidate the pathogenesis of vascular and ischemic events after vaccination**,** we examined macular vascular parameters with OCTA after each COVID-19 vaccine dose in asymptomatic individuals. We observed that a decrease in vascular density was more significant in the DCP than in the SCP when one dose of mRNA vaccine was administered after two doses of the inactivated vaccine. In accordance with these data, the DCP is more affected than the SCP in many retinal vascular events, and the reason may be the direct connection of the SCP with arterioles^(13)^.

We found a significant decrease in choriocapillaris flow in the subfoveal area. However, we also observed that flow in the 6-mm diameter submacular area was lower after vaccination than before vaccination, but this difference was not significant.

The decrease in vascular densities of the retinal capillary plexuses and choroidal flow was more evident after inactivated vaccine doses. Pichi et al. reported possible ophthalmologic manifestations, including chorioretinal ischemic disorders such as AMN and paracentral acute middle maculopathy, after receipt of an inactivated COVID-19 vaccine^(14)^. These effects have also been observed with other types of inactivated vaccines. Liu et al. showed a decrease in DCP vascular density on OCTA when AMN was developing after receipt of inactivated influenza vaccine^(15)^.

Gedik et al. studied 40 healthy individuals who received a single dose of the CoronaVac vaccine and compared OCTA images taken before and 1 week after vaccination. They also examined the vascular parameters of the macula and optic disc. They observed a general decrease in vascular densities of the SCP, DCP, and peripapillary region, but the changes were not statistically significant^(16)^. These results supported those of our study because in comparing OCTA measurements before and after the first dose of inactivated vaccine, we found a statistically insignificant decrease in macular and subfoveal choroidal flow, and this decrease reached a significant level in most macular parameters after the second dose.

Our study is the first in which macular perfusion values were measured with OCTA in individuals who received two doses of the CoronaVac vaccine and one dose of the BNT162b2 vaccine. We found no significant difference in any parameters after the second CoronaVac dose and after the BNT162b2 dose, and we found no significant effect of BNT162b2 alone on macular vascular density. However, a definite conclusion cannot be reached with these data. The participants in our study were examined after vaccination with CoronaVac twice and then BNT162b2 because this was the order in which these vaccines became available in Turkey. Further studies are necessary to evaluate the effects of the mRNA vaccine alone, without any other type of prior vaccination.

This study had two limitations: We did not account for the possibility of subclinical infections in the participants during measurement because we did not perform polymerase chain reaction tests. Also, we did not account for the possibility of circadian changes because measurements were not performed at the same time of the day at all visits. These limitations should be considered in further studies.
